# A validated spectrofluorimetric method for the determination of nifuroxazide through coumarin formation using experimental design

**DOI:** 10.1186/1752-153X-7-90

**Published:** 2013-05-23

**Authors:** Asmaa Ahmed El-Zaher, Marianne Alphonse Mahrouse

**Affiliations:** 1 Pharmaceutical Chemistry Department, Faculty of Pharmacy, Cairo University, Kasr El-Aini St, Cairo 11562, Egypt

**Keywords:** Nifuroxazide, Spectrofluorimetry, Experimental design, Coumarin, Ethylacetoacetate

## Abstract

**Background:**

Nifuroxazide (*NF*) is an oral nitrofuran antibiotic, having a wide range of bactericidal activity against gram positive and gram negative enteropathogenic organisms. It is formulated either in single form, as intestinal antiseptic or in combination with drotaverine (*DV*) for the treatment of gastroenteritis accompanied with gastrointestinal spasm. Spectrofluorimetry is a convenient and sensitive technique for pharmaceutical quality control. The new proposed spectrofluorimetric method allows its determination either in single form or in binary mixture with *DV*. Furthermore, experimental conditions were optimized using the new approach: Experimental design, which has many advantages over the old one, one variable at a time (OVAT approach).

**Results:**

A novel and sensitive spectrofluorimetric method was designed and validated for the determination of *NF* in pharmaceutical formulation. The method was based upon the formation of a highly fluorescent coumarin compound by the reaction between *NF* and ethylacetoacetate (*EAA*) using sulfuric acid as catalyst. The fluorescence was measured at 390 nm upon excitation at 340 nm. Experimental design was used to optimize experimental conditions. Volumes of *EAA* and sulfuric acid, temperature and heating time were considered the critical factors to be studied in order to establish an optimum fluorescence. Each two factors were co-tried at three levels. Regression analysis revealed good correlation between fluorescence intensity and concentration over the range 20–400 ng ml^-1^. The suggested method was successfully applied for the determination of *NF* in pure and capsule forms. The procedure was validated in terms of linearity, accuracy, precision, limit of detection and limit of quantification. The selectivity of the method was investigated by analysis of *NF* in presence of the co-mixed drug *DV* where no interference was observed. The reaction pathway was suggested and the structure of the fluorescent product was proposed. Statistical comparison between the presented method and a reported spectrophotometric one was carried out on pure and pharmaceutical formulation and revealed no significant difference.

**Conclusion:**

The proposed method was considered economic, accurate, precise and highly sensitive. It could be easily applied in laboratory quality control for the analysis of *NF* in pure form and in pharmaceutical dosage form.

## Background

**Scheme 1:**
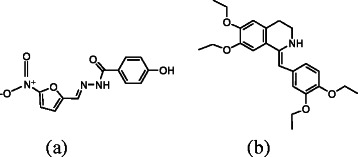
**Chemical structures of *NF *(a) and *DV *(b).**

Nifuroxazide (*NF*, Scheme 1a), chemically designated as 4-hydroxy-N'-[(5-nitrofuran-2-yl) methylidene] benzohydrazide [1], is a nitrofuran derivative with wide range of bactericidal activity against gram positive and gram negative enteropathogenic organisms. It is poorly absorbed from the gastrointestinal tract and is extensively used as an intestinal antiseptic in the treatment of colitis, acute and chronic diarrhea and gastroenteritis [2]. *NF* is an official drug in the British Pharmacopoeia [3] which recommends a potentiometric titration for its determination in pharmaceutical formulation. Various analytical techniques were reported for the analysis of *NF* including colorimetry [4,5], spectrophotometry [5-8], near IR [9], TLC [10] and HPLC [5,11]. Electrochemical methods, such as polarography [6,7,12-15] and voltammetry [14,16,17] were also applied. Only a single spectrofluorimetric method was reported for the determination of *NF* using the native fluorescence of its alkaline degradate [18]. Pharmaceutical dosage form containing a binary mixture of *NF* and drotaverine (*DV*, Scheme 1b), is well established as an intestinal antiseptic - antispasmodic formulation. Spectrophotometric, TLC and HPLC [19-21] methods were developed for the simultaneous determination of *NF* and *DV* in capsules.

Two drawbacks were found in the reported spectrofluorimetric method. First, the method could not be applied for the determination of *NF* in presence of *DV*, since the alkaline conditions required would lead to degradation of *DV*[22]. Second, the optimization of the procedure conditions was performed one variable at a time (OVAT approach) which does not guarantee at all that the real optimum will be reached. This approach would be valid only if the variables to be optimized were totally independent from each other [23]. In addition, OVAT approach requires a vast number of experiments to establish optimum levels and therefore, is time and budget consuming. These limitations can be eliminated by using experimental design (DOE) which is considered a more efficient systematic optimization technique. It has attracted attention as a multivariate optimizing approach as it takes into account the interaction among relevant factors affecting any chemical reaction. Moreover, this approach offers distinct advantages over OVAT approach, such as, the use of minimum number of experiments and feasibility of generating data that may be analyzed statistically to provide valuable information on the interactions among experimental parameters [24,25].

Based on the aforementioned observations, the main objective of this work was to develop and optimize a new spectrofluorimetric method for the determination of *NF*, based on the formation of a highly fluorescent coumarin via Von Pechmann - Duisberg condensation reaction. The optimum spectrofluorimetric conditions were estimated by a face centered composite (FCC) design using both mathematical and graphical global optimization approaches. The coumarin product was isolated and its structure was identified by IR, ^1^H-NMR and mass spectroscopy. The proposed method was tested for linearity, accuracy, intraday and interday precision. Finally, the selectivity of the suggested method was checked by the analysis of *NF* in presence of the co-mixed drug *DV*.

### Experimental

#### Instrumentation

All fluorescence measurements were carried out using a Shimadzu RF – 1501 Spectrofluorophotometer (Shimadzu Kyoto, Japan), with excitation and emission band pass of 5 nm using 1 cm quartz cell. Sonication was performed on ultrasonic processor; Soniclean 120T, 220/240v, 50/60Hz, 60W (Thebarton SA, Australia). Experimental matrices, three dimensional (3D) surface plots and contour curves were generated using Minitab (Version 15) statistical software, State College, Pennsylvania, USA. IR and MS analysis were performed at micro-analytical center, Faculty of Science, Cairo University. IR spectra were recorded using potassium bromide discs on Shimadzu IR-435 spectrometer (Shimadzu, Kyoto, Japan). Mass spectra were recorded on a Hewlett Packard 5988A GC/MS system (70ev) spectrometer (USA). ^1^H-NMR spectra were performed at national research center, on Jeol NMR Varian Gemini 500 MHZ spectrometer (Jeol, Tokyo, Japan, Varian, PaloAito, CA, USA).

#### Materials and reagents

All chemicals and solvents were of analytical reagent grade. *NF* pure sample was kindly supplied by Amoun Pharmaceutical Co., El-Obour City, Egypt. Its purity was checked according to spectrophotometric method [19] and was found to be 99.28 ± 0.745. Pharmaceutical dosage form containing *NF* was purchased from the local market. Antinal® capsules (Batch No. 112463) were labeled to contain 200 mg *NF*/capsule and drotazide® capsules (Batch No. 012034) were labeled to contain 200 mg *NF* and 40 mg *DV* /capsule. The two pharmaceutical preparations were manufactured by Amoun Pharmaceutical Co., El-Obour City, Egypt. Ethyl acetoacetate (Merck, Germany), labeled to be 98%, was used as freshly prepared 2% (v/v) solution in absolute ethanol (Sigma –Aldrich, Germany) and the solutions were discarded regularly every day. Sulfuric acid (Fischer, UK), labeled to be > 95% v/v, was used all over the work. Methanol (Riedel – de Haën, Germany) was HPLC grade. Methylene chloride (El-Nasr Pharmaceutical Chemicals, Egypt) was of analytical reagent grade.

#### Preparation of the standard solutions

##### Stock solution

*NF* stock solution was prepared by accurately weighing 10 mg of *NF* and dissolving in 20 ml absolute ethanol with the aid of sonication for 15 minutes in a 100 ml volumetric flask. The volume was completed to the mark with the same solvent to give a final concentration of 100 μg ml^-1^ of *NF*. The solution was wrapped in aluminum foil [26] and was stable for at least 7 days if kept in the refrigerator.

##### Working standard solution

A solution of final concentration of 1 μg ml^-1^ was prepared by diluting 1 ml of the standard stock solution to 100 ml using absolute ethanol.

### Experimental design for spectrofluorimetric method optimization

To optimize the critical factors affecting the reaction and explore their effects on the response, a three-level FCC design was applied. Two experimental matrices, each of 11 experiments, were designed, then experimentally performed and the corresponding fluorescence intensities were measured. The factors chosen were volume of ethyl acetoacetate (*EAA*), volume of sulfuric acid, temperature and heating time. Each factor was tried at three levels. Experimental matrix which contains the coded levels and experimental plan which reports their real values are revealed in Table 1.

**Table 1:** Experimental matrix and experimental plan of the face centered composite design

**Number of experiments**	**Experimental variables**							
	**X**_**1**_	**X**_**2**_	**X**_**3**_	**X**_**4**_	**Volume of EAA (ml)**	**Volume of H**_**2**_**SO**_**4 **_**(ml)**	**Temperature (°C)**	**Heating time (min)**
1	1	0	−1	−1	0.3	1.5	25	10
2	0	1	1	1	0.2	2.5	55	30
3	1	−1	−1	1	0.3	0.5	25	30
4	0	0	0	0	0.2	1.5	40	20
5	−1	1	0	−1	0.1	2.5	40	10
6	−1	0	−1	0	0.1	1.5	25	20
7	0	0	0	0	0.2	1.5	40	20
8	0	−1	1	0	0.2	0.5	55	20
9	1	1	1	−1	0.3	2.5	55	10
10	−1	−1	0	1	0.1	0.5	40	30
11	0	0	0	0	0.2	1.5	40	20

The FCC design supports the building of a polynomial equation which takes into account the individual, interactive (usually the interaction among more than two terms are not taken into account) and quadratic terms according to the following mathematical second-order model [27,28]:

$$Y=b_{0}+b_{1}X_{1}+b_{2}X_{2}+b_{12}X_{1}X_{2}+b_{11}X_{1}^{2}+b_{22}X_{2}^{2}$$

Where Y is the response, b_0_ is the arithmetic mean response, b_1_ and b_2_ are the regression coefficients of the factors X_1_ and X_2_, respectively and b_12_, b_11_ and b_22_ are interaction and square regression coefficients terms, respectively. The terms b_1_X_1_ and b_2_X_2_ are the individual effects of each factor, b_12_X_1_X_2_ indicates the interaction among the factors and the terms b_11_X_1_^2^, b_22_X_2_^2^ takes into account a possible non-linear (quadratic) effects of some factors.

Response surface and contour plots were constructed to evaluate the optimum conditions for the response. Plots of residuals and a lack of fit test with the analysis of variance (ANOVA) model were conducted to assure the adequacy of the model.

### Construction of calibration graph

To a set of 10 ml volumetric flasks, different aliquots equivalent to (200 - 4000 ng) from the working standard solution of the drug were quantitatively transferred and then were evaporated to dryness on a boiling water bath. The flasks were then left to cool to room temperature. *EAA* solution (0.1 ml, 2% v/v) was added to each flask followed by cautious dropwise addition of sulfuric acid (2.5 ml). The flasks were heated in a water bath adjusted at 40°C for 20 minutes, left to cool to room temperature and then the volume was completed to the mark with methanol. The fluorescence intensities of the resulting solutions were measured at 390 nm after excitation at 340 nm. The calibration graph was obtained by plotting the fluorescence intensities versus the corresponding concentrations and the regression equation was computed.

### Analysis of pharmaceutical dosage form

Accurate weights of the mixed contents of either 20 Antinal® capsules or drotazide® capsules equivalent to 10 mg of *NF* were transferred quantitatively to two 100 ml volumetric flasks and about 50 ml of absolute ethanol were added to each flask. The content of each flask was sonicated for 15 minutes, completed to volume with the same solvent, filtered and then, the first portion of the filtrate was rejected. Further dilution was done in order to obtain a working standard solution of 1 μg ml^-1^ using absolute ethanol. Different aliquots of the working standard solution were analyzed using the procedure mentioned under "Construction of calibration graph".

### Method validation

The newly developed method was validated in terms of linearity, accuracy, precision, selectivity, limit of detection (LOD) and limit of quantification (LOQ), according to the International Conference on Harmonization (ICH) Q2 (R1) guidelines [29].

#### Linearity and range

The linearity of the method was checked by analyzing five different solutions of *NF* over the concentration range 20 – 400 ng ml^-1^. Each solution was prepared and analyzed in triplicate. Calibration curve was constructed as fluorescence intensity versus the concentration of the drug and the linear relationship was determined.

#### Accuracy

The accuracy of the method was tested by analyzing solutions of the drug in triplicate at concentrations 50, 150, 250 and 350 ng ml^-1^ by using the previously mentioned experimental conditions and comparing measured and calculated values. Percentage recoveries, the mean recovery and the standard deviation (SD) were calculated. In addition, accuracy and validity of the method was determined by standard addition technique in which the recoveries of known amounts of *NF*, added to the sample solution of known concentration, were calculated.

#### Precision

The repeatability (intraday precision) was determined by replicate analysis (n=3) of standard solutions at low and high concentration levels (100 and 350 ng ml^-1^). The intermediate precision (interday precision) was conducted by repeating the analysis over a period of three consecutive working days. The overall precision of the method was expressed as percentage relative standard deviations (% RSD).

#### Selectivity

Selectivity was investigated by analyzing *NF* in pharmaceutical dosage form containing binary mixture of *NF* and *DV*. Percentage recoveries of *NF* were calculated.

#### Limit of detection and limit of quantification

LOD and LOQ of the developed method for the analysis of *NF* were established according to (ICH) Q2 (R1) guidelines, based on the standard deviation of the response and the slope, using the following equation:

LOD=3.3×SD/slope⋯⋯⋯LOQ=10×SD/slope

## Results and discussion

Compared to absorption spectroscopic methods, the most attractive features of spectrofluorimetric analysis are high sensitivity and high selectivity. Its sensitivity is one to three orders of magnitude better than absorption spectroscopy while its greater selectivity is based on the fact that fewer substances fluoresce than absorb radiation [30,31]. Another advantage is the large linear concentration range of spectrofluorimetric methods which is greater than those encountered in absorption spectroscopy [32].

### Optimization of reaction conditions

OVAT approach, which was traditionally employed for method development, generally requires a large number of experimental runs. In addition, it does not include interaction term and thus it does not depict the collective effects of various factors on the response [33]. To overcome this problem, the optimal reaction conditions were defined by applying DOE, in which factors are varied together. It is a statistical technique for quickly optimizing performance of systems, with two general issues, first, designing an optimal experiment and second, analyzing its results [24,28].

#### Preliminary study and factor selection

Some studies were carried out prior to the optimization by DOE. Excitation and emission wavelengths were optimized by searching the maximum fluorescence intensity by means of the recorded spectra. The excitation and emission optimal wavelengths were 340 nm and 390 nm, respectively. Several solvents were tested for the fluorescent measurements (methanol, ethanol, acetonitrile and water) and maximum fluorescence intensity was obtained with methanol. Preliminary investigations reveal that four factors could be relevant and affect the reaction. Volumes of *EAA* (X_1_), volume of sulfuric acid (X_2_), temperature (X_3_) and heating time (X_4_) were considered the critical factors to be studied in order to establish an optimum fluorescence.

#### Face centered composite design

Factorial design, in which factors are set at two levels, assumes that the effect of factors on the response is linear (first-order factor effects). Since the yield of the chemical reaction is a function of time and temperature which are usually related in a curvilinear fashion to the resultant yield, center-point (the mean value of a factor) should be added in the center of the design, resulting in star design. The face centered composite design (FCC) is a special case of central composite design and is composed by a factorial design and a star design. It was utilized to evaluate the main, interaction and quadratic effects of the studied factors on the response [23,28].

**Figure 1:**
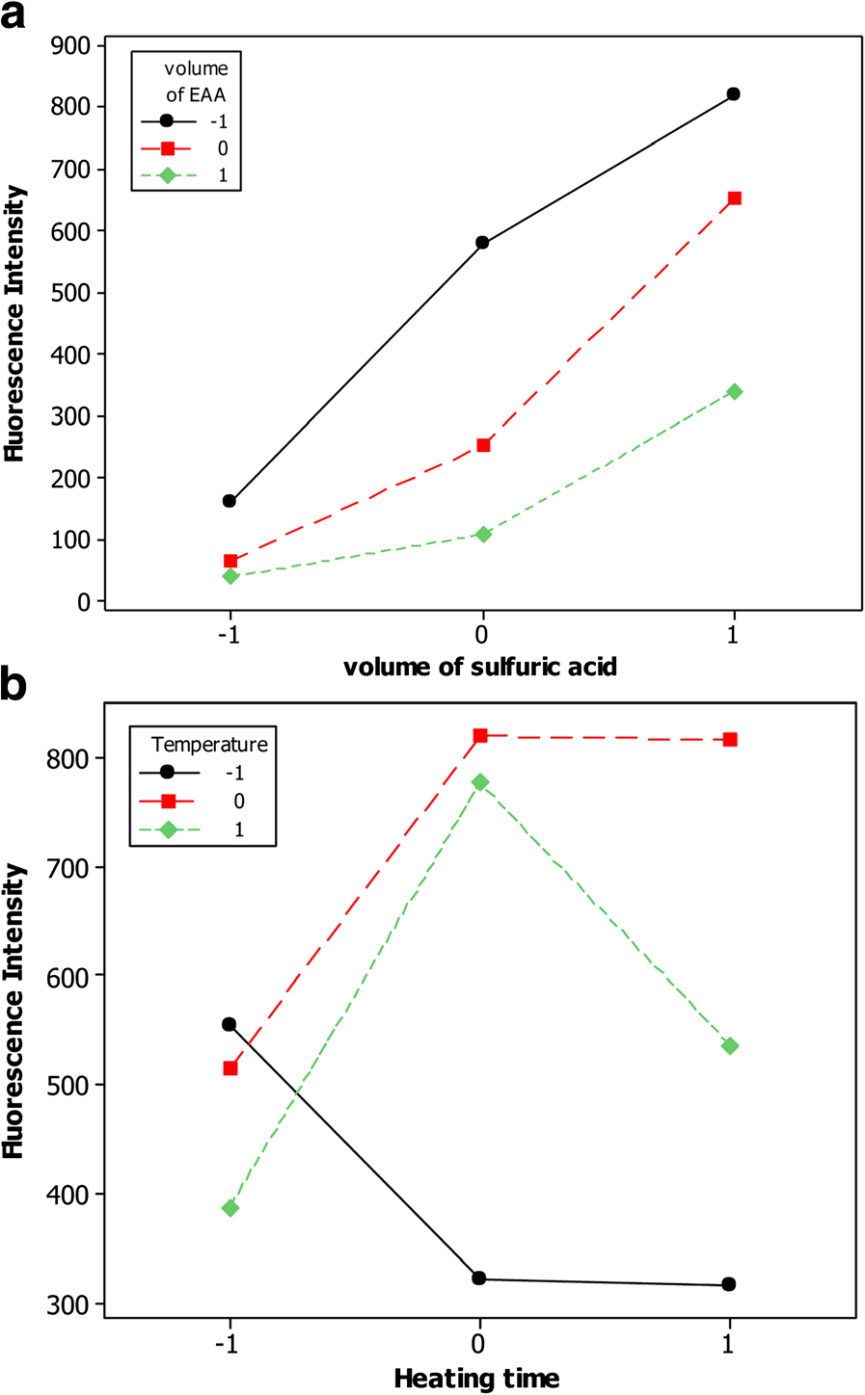
**Interaction plots for the effect of volume of *EAA *and volume of H**_**2**_**SO**_**4 **_**(a) and the effect of temperature and heating time (b) on fluorescence intensity.**

Experimental matrix of FCC was built using each two factors at three levels. The number of experimental runs was 2^K^ + 2K + 1, where K was the number of factors. The first 2^K^ runs were the same as factorial design, the next 2K axial experiments were symmetrically spaced at ± 1 along each variable axis and at least one central point. Two to five center repetitions were generally carried out in order to know the experimental variance and to test the predictive validity of the model [34]. Therefore, a two- factor FCC design required 11 runs (with two center repetitions) while a four-factor FCC design required 27 runs. For this reason, in order to decrease number of experiments, the four critical factors of the reaction were divided into two separate models. Each two factors were chosen based on the interaction plots, Figure 1, where the relative slopes of the lines depicted the presence of interaction between (X_1_ , X_2_) and (X_3_ , X_4_). The factors were tried at three levels and levels were coded for simplification, -1 for the minimum value, +1 for the maximum value and 0 for the central one. Accordingly, two experimental matrices, each of 11 experiments, were designed and all experiments were performed in randomized order to minimize any bias on the response due to uncontrolled factors [24]. Experimental matrix and plan were reported in Table 1.

The coefficients of the second–order polynomial model were computed and the following equation models were obtained:

(1) $$\begin{array}{l} Y=159.48-75.37X_{1}\\ \;+145.62X_{2}-88.28X_{1}X_{2}+42.99X_{1}^{2}\\ \;+132.56X_{2}^{2} \end{array}$$

(2) $$\begin{array}{l} Y=701.72+193.67X_{3}\\ \;+47.41\;X_{4}-34.58X_{3}X_{4}-203.80X_{3}^{2}-2.75X_{4}^{2} \end{array}$$

Where Y is the fluorescence intensity, X_1_, X_2_, X_3_, and X_4_ are volume of EAA, volume of sulfuric acid, temperature and heating time, respectively. By simply replacing X_1_, X_2_, X_3_, and X_4_, it will be possible to predict the response for any possible setting, even for those experiments that have not been actually performed.

Equations (1, 2) reveal that the fluorescence intensity is inversely related to the volume of EAA. While the increase in volume of sulfuric acid and temperature significantly increases the fluorescence intensity. However, the highly significant quadratic terms ($X_{2}^{2}$ and $X_{3}^{2}$) indicate a possible curvature and non-linear correlation between the factor and the output of the equation. The individual effects of temperature and heating time are positive and their quadratic effects are negative thus indicating that the fluorescence intensity increases with increasing of the factor up to a critical threshold after which a further increase results in a decrease of the response (0 level of X_3_ and X_4_ were chosen). In addition, the significant interaction between volumes of EAA and sulfuric acid and that of temperature and heating time decreases the fluorescence. Another interesting idea can be inferred from the interactive terms, where the negative sign suggests that each two factors act in a negative way each other, i.e. to increase the response, volume of EAA is maintained at low level while increasing that of sulfuric acid. Regression Table 2 reveals the values of regression coefficients and their associated *p*-values, which are used to determine which of the effects in the model are statistically significant. It was observed that all the four factors and X_1_ X_2_ interaction significantly affect the fluorescence intensity (*p* < 0.05), i.e. the fluorescence intensity differs depending on the four factors and the effect of volume of EAA on the response depends on the volume of sulfuric acid. Significant quadratic effect $X_{3}^{2}$ assures that the relationship between volume of sulfuric acid and fluorescence intensity followed a curved line rather than a straight line. However, X_3_ X_4_ interaction and the quadratic models $X_{1}^{2}$ and $X_{4}^{2}$ did not have a significant effect on the response (*p* > 0.05).

**Table 2:** **Estimated regression coefficients and associated probability values ( *p *-value) for fluorescence intensity**

**Equation (1)**			**Equation (2)**		
**Term**	**Coefficients**	***p***	**Term**	**Coefficients**	***p***
Constant	159.48	0.001	Constant	701.72	0.000
Volume of EAA	−75.37	0.023	Temperature	193.67	0.000
Volume of sulfuric acid	145.62	0.001	Heating time	47.41	0.035
Volume of EAA * Volume of EAA	42.99	0.299	Temperature * Temperature	−203.80	0.000
Volume of sulfuric acid * Volume of sulfuric acid	132.56	0.011	Heating time * Heating time	−2.75	0.921
Volume of EAA* Volume of sulfuric acid	−88.28	0.028	Temperature * Heating time	−34.58	0.165

**Figure 2:**
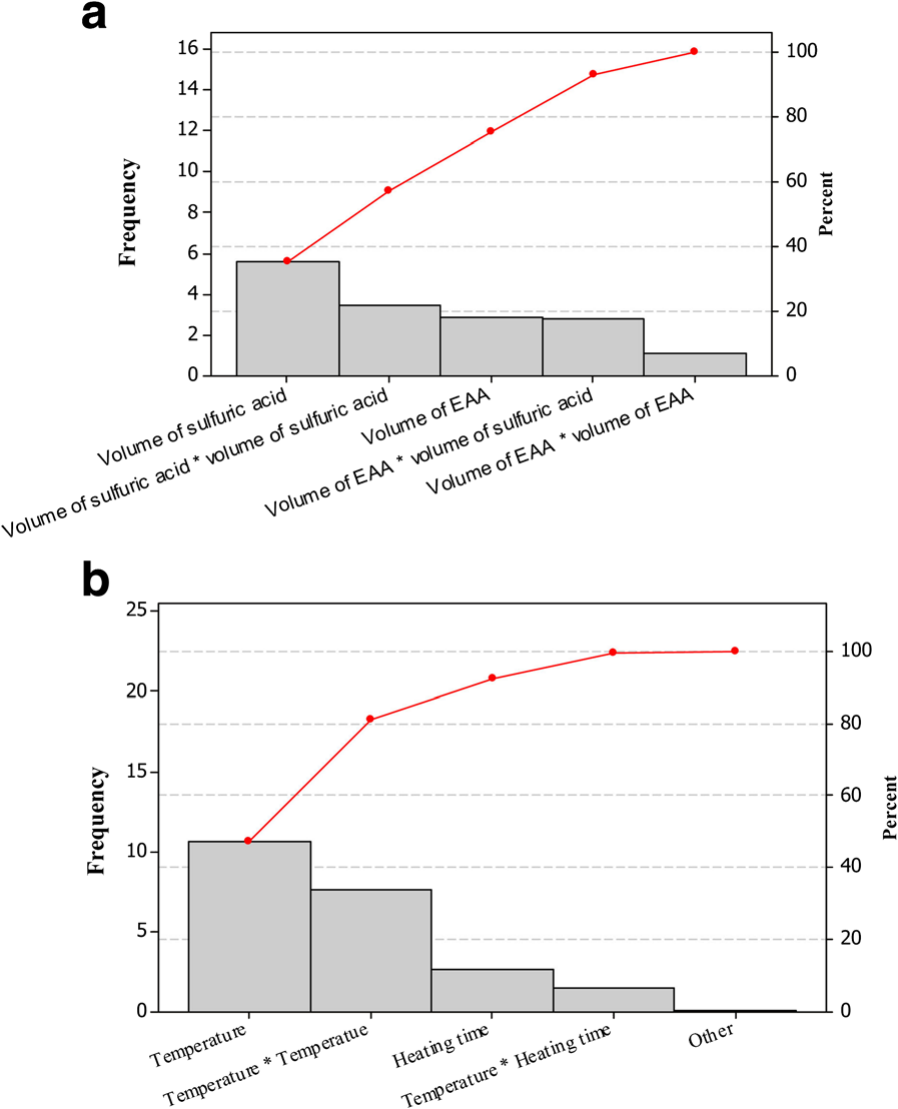
Pareto charts showing the influence of volume of sulfuric acid and volume of EAA (a) and that of temperature and heating time (b) on the fluorescence intensity.

Pareto charts, Figure 2, reveal the factors that were statistically significant (*p* < 0.05). It contains a bar for each factor whose length is proportional to its corresponding effect on the reaction [35]. Each factor is significant as individual, moreover, the statistical analysis highlights a significant effect of the interaction of volume of EAA and that of sulfuric acid.

#### Graphical evaluation of FCC design

**Figure 3:**
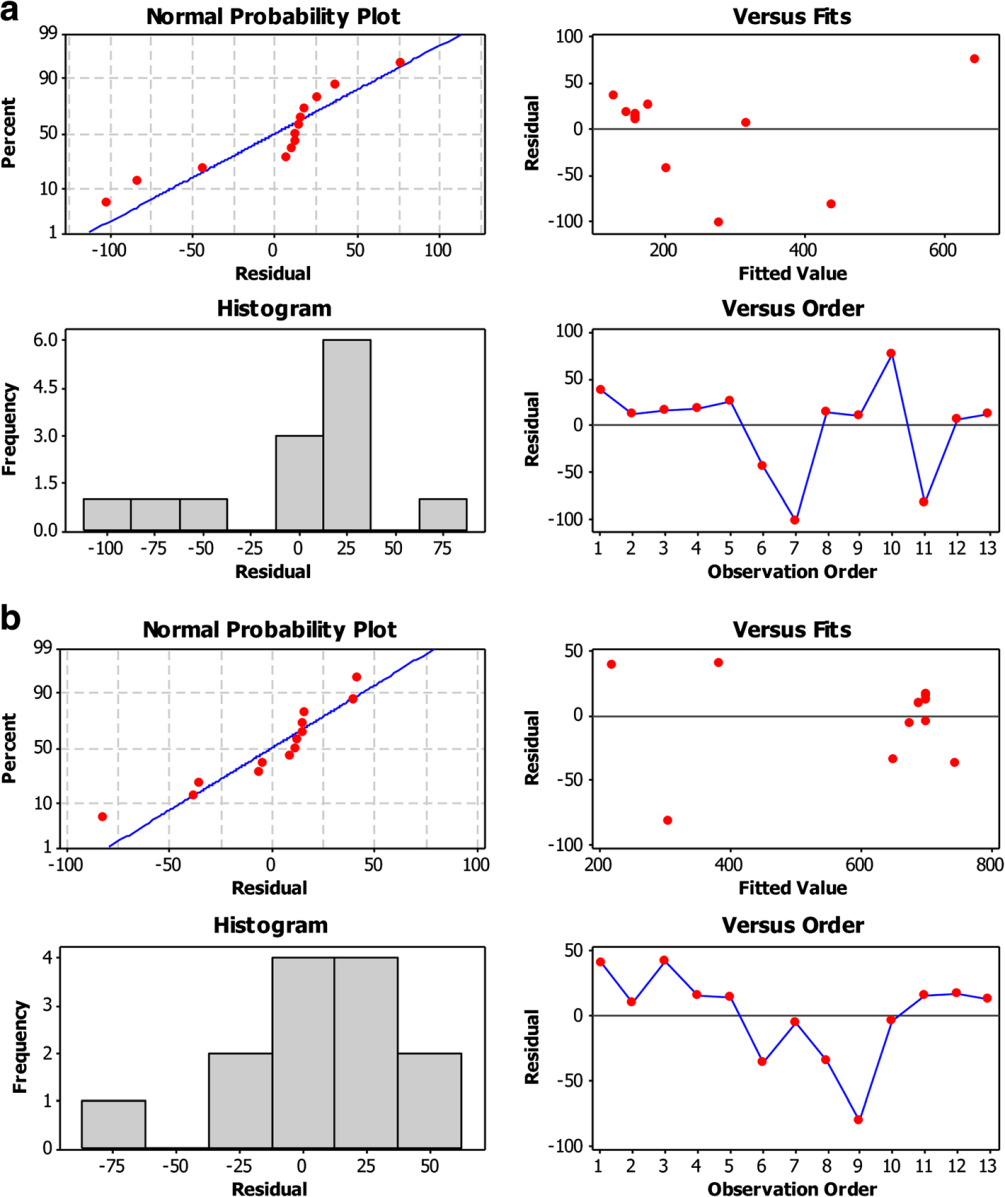
**Response surface plots (a) and contour plots (b) showing the effect of volume of EAA, volume of sulfuric acid (a**_**1**_**, b**_**1**_**) and temperature and heating time (a**_**2**_**, b**_**2**_**) on fluorescence intensity.**

**Figure 4:**
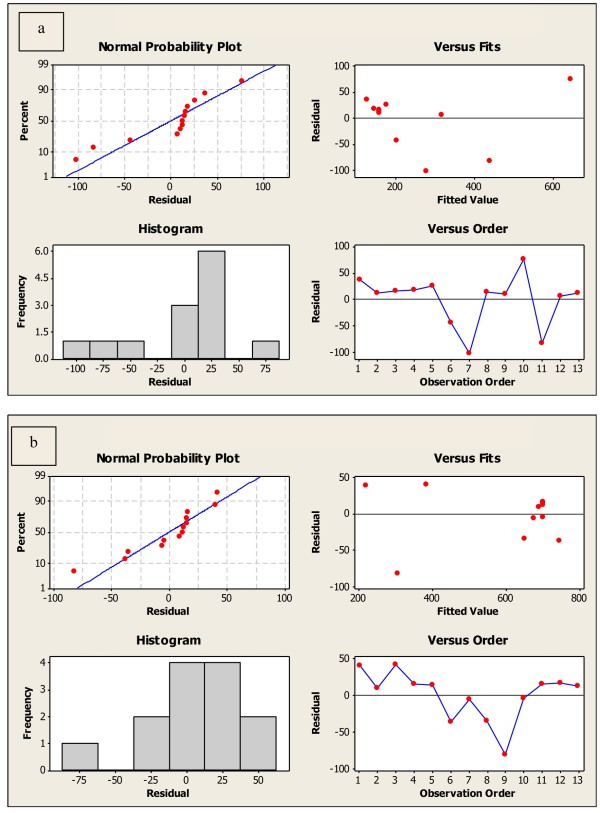
Normal probability plot, histogram, residuals versus fits and residuals versus order for fluorescence intensity, model 1 (a) and model 2 (b).

Polynomial equations were graphically represented by a response surface plot which is a 3D plot reporting the interaction of two factors and their effect on the equation output, the target optimum is the top of the mountain. A modification of the surface response plot is the contour plot, reporting the interactions in a two-dimensional (2D) figure, the target optimum is close to the centre of the contours [24]. Response surface and contour plots, related to each model, were analyzed to visualize the parameters and their interactions on the response. Figure 3 confirm the results predicted from the mathematical equations, optimal conditions for the suggested procedure were also deduced. Surface plot, Figure 4a_1_, reveals that the highest fluorescence intensity was obtained when the volume of EAA was 0.1 ml and that of sulfuric was 2.5 ml. In addition, Figure 4a_2_ reveals a maximal response value which corresponds to 40°C temperature and 20 min heating time. Contour plots, Figure 4b_1_ and 4b_2_, show curvature indicating the non-linear effects of these factors on fluorescence intensity. Contour plots assist the prediction of response at any area of the experimental domain [24].

#### Residual analysis

Residuals are the deviations of the observed values of the response from the predicted values, given the current model. Examination of residuals assists in checking model adequacy [28]. Residual plots are used to evaluate the data for nonnormality, nonrandom variation, nonconstant variance and outliers. Normal plot of the residuals reveals that the residuals follow a straight line, which indicates that the errors are normally distributed, Figure 4. Histogram of the residuals is bell-shaped indicating the absence of skewness and outliers. Based on residuals versus fits plot, the residuals appear to be randomly scattered about zero, therefore, there is no evidence of nonconstant variance. The residuals in the residuals versus order plot fluctuate in a random pattern around the center line and hence no evidence exists that the error terms are correlated with one another [25], Figure 4. Therefore, residual analysis supports the fact that the model fits the data adequately.

#### Statistical analysis of the model

Along with the polynomial equation, the main outputs of a FCC design are some statistical parameters that measure the goodness of fitting of the proposed equation. The model was validated by ANOVA, Table 3. In ANOVA analysis, a significant model and a non significant lack of fit are desired as a model that fits the data is essential for optimization studies [25]. The regression models are significant (*p* = 0.002, 0.000), that is, at least one of the terms in the regression equation makes a significant impact on the mean response. The *p*-value for the squared effects is less than 0.05. Therefore, there is a significant quadratic effect, that is, the relationship between $X_{2}^{2}$ and $X_{3}^{2}$ and yield does not follow a straight line but rather a curved line. The *p*-value of 0.028 for the volume of EAA by volume of sulfuric acid interaction implies a significant interaction effect, that is, the effect of volume of EAA on reaction yield depends on volume of sulfuric acid. On the other hand, temperature and heating time interaction is non-significant (*p* = 0.165). Although the model exhibits highly significant factor effects, Lack-of-fit is also highly significant. Such a situation often arises if the model fits the data well and if the measurement process is highly precise [27]. Therefore, the models that have been developed can be used to predict the fluorescence intensity of *NF* within the limits of the experiment [25].

**Table 3:** Analysis of variance (ANOVA) results for fluorescence intensity

**Model (1)**		**Model (2)**	
**Source**	***p***	**Source**	***p***
Regression	0.002	Regression	0.000
Linear	0.001	Linear	0.000
Square	0.010	Square	0.000
Interaction	0.028	Interaction	0.165
Residual error		Residual error	
Lack-of-fit	0.000	Lack-of-fit	0.000

### Reaction mechanism

**Scheme 2:**
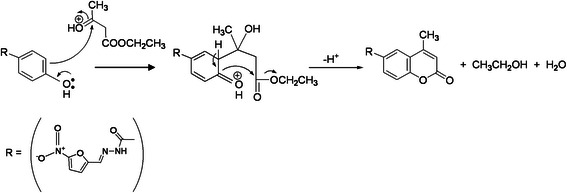
**Suggested pathway for the reaction between *NF *and *EAA*/ H**_**2**_**SO**_**4**_**.**

**Figure 5:**
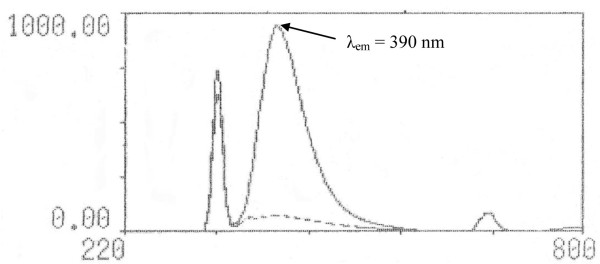
**Excitation and emission spectra of the coumarin product produced by reaction between 350 ng ml**^**-1 **^**of *NF *and *EAA *in methanol ( ____ ) and reagent blank (− − − -) (λ**_**ex **_**= 340 nm, λ**_**em **_**= 390 nm ).**

Upon optimization of the factors affecting the fluorescence intensity, the spectrofluorimetric method was applied to the determination of *NF* in pure form and in capsules. The procedure consists in condensation of the phenolic derivative with β-ketoester in presence of excess acid as catalyst, leading to the formation of a β-hydroxyester [36]. By analogy to previous reports, the reaction was proposed to proceed as shown in Scheme 2. *NF* (bearing a phenolic group) readily coupled with EAA, in presence of sulfuric acid as dehydrating agent, producing a yellow highly fluorescent coumarin compound, which exhibited a fluorescence maximum at 390 nm when excited at 340 nm, Figure 5. The coumarin derivative was formed immediately and was stable for at least two hours. The Von Pechmann-Duisberg condensation proved to be efficient and simple reaction used to produce fluorescent coumarins. It was found that addition of EAA and sulfuric acid to different aliquots of *NF* working standard solution in absolute ethanol leads to non reproducible results. It was thought that variation of volume of absolute ethanol from one aliquot to another was the reason for the non reproducibility. Therefore, the aliquots were evaporated to dryness on a boiling water bath and then, cooled before adding EAA and sulfuric acid, thus leading to reproducible results.

### Isolation and identification of the coumarin product

To 0.5 gm *NF*, EAA (3 ml) and sulfuric acid (10 ml) were added. The mixture was heated in a water bath at 40°C for 20 min. The reaction mixture was cooled, then poured on crushed ice and stirred for 15 minutes. The product was extracted from the reaction mixture twice with methylene chloride (50 x 2). After extraction, the organic layer was left to evaporate and the obtained concentrated extract was collected. The assignment of the formed coumarin was based on the comparison of the IR and ^1^H NMR spectral data with those of the intact drug. The IR spectrum of the coumarin product revealed the disappearance of the phenolic band at 3361.32 cm^-1^ in the IR spectrum of *NF*, meanwhile, the appearance of the lactone C=O band at 1729.8 cm^-1^. The ^1^H-NMR spectrum of the coumarin product revealed the appearance of a singlet signal at δ 2.1387 ppm, integrated for three protons due to CH_3_ group and a singlet signal at δ 6.1112 ppm, integrated for one proton due to CH of the lactone ring. Moreover, the molecular weight of the coumarin product was confirmed by mass spectrum which revealed the molecular ion peak (M^+^) at 341.

### Method validation

The optimized spectrofluorimetric method was validated according to (ICH) Q2 (R1) guidelines [29].

### Linearity and range

Applying the procedure mentioned under construction of a calibration graph, the relationship between *NF* concentration and fluorescence intensity was found to be linear. The good linear relationship was revealed by the high value of the correlation coefficient. Descriptive statistics of the regression line showed low values of standard error of estimation, standard error of slope (*S*_*b*_) and that of intercept (*S*_*a*_) which revealed high accuracy with minimum deviations and low scattering of the calibration points, Table 4.

**Table 4:** **Assay parameters and method validation obtained by applying spectrofluorimetric method for the determination of *NF***

**Parameter**	***NF***
Excitation wavelength	340 nm
Emission wavelength	390 nm
Range of linearity	20 - 400 ng ml^-1^
Regression equation	y = 1448.447x + 101.590
Correlation coefficient (*r*)	0.9997
*S*_*b*_	14.185
*S*_*a*_	3.477
Confidence limit of the slope	1448.447 ± 45.137
Confidence limit of the intercept	101.590 ± 11.064
Standard error of the estimation	4.310
LOD^a^	0.01 ng ml^-1^
LOQ^a^	0.03 ng ml^-1^
Intraday^b^	
% RSD	0.692 – 0.598
Interday^c^	
% RSD	0.688 – 0.395

^a^ Limits of detection and quantification are determined via calculations [29]:

LOD= 3.3×SD/slope LOQ= 10×SD/slope, where SD is standard deviation of response.

^b^ The intraday (*n* = 3), average of two concentrations of NF (100, 350 ng ml^−1^), repeated three times within the day.

^c^ The interday (*n* = 3), average of two concentrations of NF (100, 350 ng ml^−1^), repeated three times in three successive days.

### Accuracy

Accuracy of the analytical procedure is the closeness of agreement between a true value and the value obtained. Table 5 reveals that the SD values for all the samples were less than 1% suggesting that the method was accurate.

**Table 5:** **Application of the proposed spectrofluorimetric method for the determination of *NF *in pure samples**

**Claimed taken (ng ml**^**-1**^**)**	**Claimed found (ng ml**^**-1**^**)**	**% Recovery**^**a**^
50	49.991	99.98
150	150.009	100.07
250	250.896	100.36
350	347.552	99.30
Mean		99.93
± SD		0.449

^a^ Average of three determinations.

Furthermore, standard addition technique showed satisfactory results as the percentage recoveries achieved ranged between 98.38 and 100.97% and the corresponding SD were well below 1%, indicating that the method was accurate and valid, Table 6.

**Table 6:** **Determination of *NF *in capsules by the proposed spectrofluorimetric method and application of standard addition technique**

**Antinal® capsules**				**Drotazide®capsules**			
**Claimed (ng ml**^**-1**^**)**	**% Recovery**^**a**^	**Pure *NF *added (ng ml**^**-1**^**)**	**% Recovery**^**a**^	**Claimed (ng ml**^**-1**^**)**	**% Recovery**^**a**^	**Pure *NF *added (ng ml**^**-1**^**)**	**% Recovery**^**a**^
100	99.70	80	100.11	100	99.01	80	100.97
		100	100.80			100	98.73
		120	100.68			120	99.53
150	100.53	120	100.11	150	100.07	120	100.11
		150	100.34			150	100.80
		170	99.90			170	99.50
200	99.56	160	98.38	200	100.25	160	100.11
		180	99.72			180	100.87
		200	99.42			200	99.42
Mean	99.93		99.94		99.78		100.00
± SD	0.524		0.730		0.670		0.773

^a^ Average of three different determinations.

### Precision

Results for the determination of repeatability and intermediate precision are displayed in Table 4. Low values of % RSD confirmed that the suggested method was precise, has good repeatability and hence, was satisfactory for quality control measurements.

### Selectivity

Selectivity is the ability of the analytical method to measure the analyte response in presence of interferences. It was ascertained by applying the method to the pharmaceutical dosage form containing the co-formulated drug *DV*. Results in Table 6 reveal that no interference was observed either from *DV* or from the frequently encountered excipients, indicating that the proposed method was highly selective for the analysis of *NF*. Moreover, results close to 100% were obtained for the determination of NF in dosage form, Table 6. These results confirm the selectivity of the method.

### Limit of detection and limit of quantification

The LOD obtained with the developed method was 0.01 ng ml^-1^, as shown in Table 4. Therefore, the proposed method was found to be more sensitive than the reported spectrofluorimetric one for the determination of *NF*[18] whose LOD was 0.008 μg ml^-1^.

### Statistical analysis

A reference spectrophotometric method was adopted for the analysis of *NF* in pure form and in pharmaceutical preparation [19]. Statistical comparison of the results obtained by the proposed and reported methods is shown in Table 7. The calculated *t*- and *F*- values are less than the theoretical ones indicating that there is no significant difference between the proposed method and the reported one with respect to accuracy and precision.

**Table 7:** Statistical analysis of the results obtained by applying the proposed spectrofluorimetric method and the reference method

***NF***	**Item**	**Spectrofluorimetry**	**Reference method [**[19]**]**
Pure form	Mean	99.93	99.28
	SD	0.449	0.745
	RSD	0.449	0.750
	*n*	4	5
	Variance	0.201	0.555
	*t-*value	0.05 (2.365)*	
	*F-*value	2.761 (9.12)*	
Antinal® capsules	Mean	99.93	100.49
	SD	0.524	0.955
	RSD	0.524	0.950
	*n*	3	5
	Variance	0.275	0.912
	*t-*value	1.07 (2.447)*	
	*F-*value	3.316 (19.25)*	

* Figures in parentheses are the corresponding theoretical *t* -and *F-*values at *p* = 0.05.

## Conclusion

A systematic and practical approach was employed to develop an efficient and selective spectrofluorimetric method to quantify *NF* in pure and capsule forms. Experimental design was successfully applied to effectively evaluate the main effects of factors that significantly affected the fluorescence intensity and to determine their interactions and quadratic effects with the least number of runs. The application of response surface methodology with face centered composite design to modeling and optimizing the performance of the suggested method proved to be an economic approach for extracting large amount of information while saving time and cost. After optimization, all the performed response surface, contour plots, plots of residuals, lack of fit test and ANOVA results support each other and confirm the adequacy of the model. The method was validated and the obtained results indicate good linearity and reproducibility. The method was further compared with a reported UV method and proved to be more sensitive and selective. The proposed method is accurate, specific, and sensitive so can be applied for the routine analysis of *NF* in pure form and in pharmaceutical dosage form, either alone or in presence of *DV*.

## Abbreviations

NF: Nifuroxazide; DV: Drotaverine; OVAT: One variable at a time; DOE: Experimental design; FCC design: Face centered composite design; EAA: Ethylacetoacetate; ANOVA: Analysis of variance; ICH: The International Conference on Harmonization; SD: Standard deviation; RSD: Relative standard deviation; LOD: Limit of detection; LOQ: Limit of quantification.

## Competing interests

The authors declare that they have no conflict of interests.

## Authors’ contributions

AAZ collected the literature review, proposed and explained the experimental design work, revised the manuscript critically, participated in the results, assay validation and discussion. MAM proposed the analytical method, helped in understanding DOE, performed the experimental design work using Minitab software, analyzed the data statistically and prepared the draft version of the manuscript. Both authors conducted the optimization of the assay conditions, carried out the analytical experimental work, read and approved the final manuscript.
